# Viewpoint: a response to “Screening and isolation to control methicillin-resistant *Staphylococcus aureus*: sense, nonsense, and evidence”

**DOI:** 10.1186/s13756-015-0044-9

**Published:** 2015-02-05

**Authors:** Kevin T Kavanagh, Lindsay E Calderon, Daniel M Saman

**Affiliations:** Health Watch USA, 3396 Woodhaven Dr, P.O. Box 1403, Somerset, KY 42503 USA; Eastern Kentucky University, Richmond, KY USA; Essentia Institute of Rural Health, Duluth, MN USA

**Keywords:** Methicillin-resistant staphylococcus aureus, Surveillance, ADI, Chlorhexidine, Research integrity, Active detection and isolation, MRSA, CLABSI

## Abstract

Surveillance and isolation for the prevention of methicillin-resistant Staphylococcus aureus (MRSA) has become a controversial topic, one that causes heated debate and appears to be surrounded by both politics and industrial conflicts-of-interest. There have been calls from numerous authors for a movement away from rigid mandates and toward an evidence-based medicine approach. However, much of the evidence can be viewed with an entirely different interpretation. Two major studies with negative findings have had an adverse impact on recommendations regarding active detection and isolation (ADI) for MRSA. However the negative findings in these studies can be explained by shortcomings in study implementation rather than the ineffectiveness of ADI. The use of daily chlorhexidine bathing has also been proposed as an alternative to ADI in ICU settings. There are shortcomings regarding the evidence in the literature concerning the effectiveness of daily chlorhexidine bathing. One of the major concerns with universal daily chlorhexidine bathing is the development of bacterial resistance. The use of surveillance and isolation to address epidemics and common dangerous pathogens should solely depend upon surveillance and isolation’s ability to prevent further spread to and infection of other patients through indirect contact. At present, there is a preponderance of evidence in the literature to support continuing use of surveillance and isolation to prevent the spread of MRSA.

## Review

### Introduction

Surveillance and isolation for the prevention of methicillin-resistant *Staphylococcus aureus* (MRSA) has become a controversial topic, one that causes heated debate and appears to be surrounded by both politics and industrial conflicts-of-interest. As pointed out by the editorial by Fätkenheuer, et al. [1], active detection and isolation (ADI) for MRSA requires significant staff resources. These are resources that cost-driven institutions may be reluctant to allocate [2].

There have been calls from numerous authors [3] along with a recent Lancet editorial by Fätkenheuer, et al. [1] for a movement away from rigid mandates and toward an evidence-based medicine approach. However, much of the evidence can be viewed with an entirely different interpretation; with many advocating not to set standards and for a “one size does not fit all’ mentality. The objective of this article is to offer a counter-opinion by reassessing the literature as quoted by Fätkenheuer, et al. [1], and along with other studies put forth an alternate evidence based conclusion.

### Active detection and isolation

Fätkenheuer, et al. [1] discuss negative findings from two studies [4,5] that can readily be explained by shortcomings in study implementation rather than the ineffectiveness of ADI [3,6,7]. In two additional studies [8,9] involving chlorhexidine bathing, the negative results regarding the use of ADI may have been overstated, in that ADI was not demonstrated to be ineffective. Taken together, whether ADI is less effective than chlorhexidine body washes depends upon data interpretation [6,7].

A major study that reported failure of ADI was by Harbarth, et al. [4], who found that well over half of the patients who underwent preoperative surveillance and were known to have MRSA prior to the operation were not given effective perioperative antibiotics against MRSA. Additionally, in 31% of the surveillance group, the positive test results were not available until after surgery. Thus, in these latter patients, intervention was not possible. Finally, bias was introduced in this study, along with a questionable deviation from the standard of care, with the report that ten patients in the study who developed a MRSA surgical site infection were known carriers prior to surgery and were not given prophylaxis effective against MRSA.

The next highly cited study that reported the failure of surveillance to reduce MRSA colonization or infection was that of Huskins, et al. [5] However, in this study, the results of admission cultures from the experimental group were not available for five days [10], and there was suboptimal staff compliance with contact precaution protocols. For example, gloves were only used in a median of 82% of cases, gowns 77%, and hand hygiene 69% of the time.

In contradistinction, there have been three studies that have had rigorous controls supporting ADI, those by Robicsek, et al. [11], Rodriguez-Bano, et al. [12], and Lee, et al. [13] In addition to isolation, Rodriguez-Bano, et al. [12] and Lee, et al. [13] also decolonized their patients. Lee, et al. [13] also observed that, except in clean surgical wards, ADI and decolonization alone were not effective, nor was enhanced hand hygiene. These interventions had to be coupled together before a significant reduction in MRSA infections was observed.

As discussed in previous reviews [3,6,7], there have also been many other studies supporting ADI which relied on pre-post designs that have been criticized for not having concurrent control groups that would detect any changes over time (secular trends). However, it is possible that the bias introduced by secular trends can be in either direction. MRSA infections may increase over time due to an increased load on a facility of colonized and infected patients from the community or decrease due to implementation of seemingly unrelated infection control protocols in the facility. Nearly all of these studies have shown a positive effect in MRSA reduction, with the reduction in many studies reaching statistical significance [3,6,7]. The Veterans Health Administration (VA) has performed large scale studies with reduced rates in MRSA infections in hospitals [14] and nursing homes [15] using a bundle approach incorporating ADI. However, a VA study by Jain, el al [14]. has been criticized for not controlling for secular trends. Is spite of its limitations, the Jaine, et al. [14], study included almost two million admissions with over eight million patient days and achieved some of the best control rates for MRSA in the United States.

### Chlorhexidine body washes

The use of daily unit-wide chlorhexidine bathing has been proposed as an alternative to surveillance and isolation [16]. Two major studies by Huang, et al. (REDUCED MRSA study) [8], and Derde, et al. [9] found this infectious disease control measure to be efficacious. However, we disagree with Fätkenheuer, et al. [1] in that neither study evaluated ADI for MRSA as compared to a no intervention control, and thus, did not show ADI to be “Not Effective”.

The surveillance and isolation group in the REDUCED MRSA study did not show a reduction in MRSA clinical isolates between the ADI baseline and ADI intervention period. However, the study’s methodology revealed that patients in both the baseline and intervention period underwent ADI. Thus, the comparison between the baseline and intervention arm was to control for secular trends, not to evaluate ADI.

The REDUCED MRSA study did show a significant reduction in MRSA clinical isolates with unit-wide chlorhexidine bathing compared to ADI, but the reduction in MRSA bacteremia did not reach significance. The largest area of significant reduction was with a reduction in infections in the “Any Pathogen” metric, but the organisms that accounted for the vast majority of the reduced infections were skin commensal bacteria. However, it should be noted that although “commensal bacteria” were not the targeted organisms, they can still cause dangerous bloodstream infections, especially in immunocompromised patients.

Derde, et al. [9] also showed a reduction in MRSA acquisition with improved patient hygiene (enhanced hand hygiene plus daily chlorhexidine body washing). The addition of ADI to the improved patient hygiene protocol did not provide an additional reduction in MRSA acquisition. We agree with Fätkenheuer, et al. [1] that the effectiveness attributable to the bundle but disagree that ADI was shown to be ineffective. There was no comparison of ADI used alone to a non-intervention or to a non-chlorhexidine body washing group. Thus, this study does not preclude the effectiveness of ADI used alone.

Neither study had a detergent bathing control, which is important in light of the work of Rotter, et al. [17] who found that preoperative bathing with chlorhexidine did not reduce surgical infections compared to bathing with a detergent. Climo, et al. [18] compared reduction in primary bloodstream infections with daily bathing using 2% chlorhexidine gluconate washcloths to daily bathing with nonantimicrobial washcloths. They did not observe a significant decrease in MRSA or vancomycin-resistant enterococcus (VRE) bloodstream infections in the chlorhexidine group compared to the nonantimicrobial washcloth group, possibly related to sample size. They did observe a decrease in central line associated bloodstream infections (CLABSIs) and multi-resistant drug organism acquisitions. The most prominent decrease in bloodstream infections was for coagulase-negative staphylococci.

Not all studies have been supportive of the efficacy of chlorhexidine. In a literature review evaluating preoperative skin antisepsis using chlorhexidine, Maiwald and Chan [19] could find “no evidence that chlorhexidine without alcohol was effective” regarding the prevention of bloodstream or surgical infections. Moreover, many trials compared chlorhexidine plus alcohol (two antiseptics) to povidone-iodine alone (one antiseptic) with several trials then attributing the clinical efficiency to chlorhexidine alone [19]. Although skin antisepsis for the prep of an operative incisional site is different from daily bathing, one can argue that a surgical prep is a more methodical and intense localized application of the antiseptic and, thus, would be expected to have increased efficacy.

In addition, one major concern with universal daily chlorhexidine bathing is the production of bacterial resistance. Genes for reduced susceptibility were observed by Derde, et al. [9] and the incidence showed a slight non-significant progression between Phase I and Phase III of their trials (14 of 110 isolates to 16 of 113 isolates). The Center for Disease Dynamics, Economics & Policy has shown that it may take decades for resistance to develop and may not be detected in any single trial [20]. Of greater concern are the recent observations by Suwantarat, et al. [21] who found that patients who were bathed daily with chlorhexidine were more likely to have CLABSI caused by organisms that had decreased susceptibility to chlorhexidine, and by Lee, et al. [22] who observed chlorhexidine resistance independently predicted MRSA decolonization failure.

### Industrial conflicts-of-interest

Concerns over industrial influence with the United States’ infectious disease policy was heightened with the controversy surrounding Dr. Charles Denham. Several authors have suggested [23,24] that Dr. Denham may have used the study by Darouiche, et al. [25] which evaluated chlorhexidine-alcohol versus povidone iodine to influence National Quality Forum (NQF) recommendations regarding antiseptics, promoting the use of CareFusion’s ChloraPrep formulation. According to Policy & Medicine [24] and Mass Devices [26], the Darouiche, et al. [25] study was funded by Cardinal Health (later CareFusion) and one of the authors was a CareFusion employee. In addition, Dr. Denham was co-chair of the NQF Safe Practices Committee. The Centers for Medicare & Medicaid Services (CMS) has contracted with NQF to develop metrics and make recommendations regarding patient safety, to be used in CMS’s value purchasing initiatives.

On Jan. 9, 2014, CareFusion entered into a 40.1 million dollar settlement with the United States Department of Justice to settle allegations that included 11 million dollars in kickbacks, allegedly given to Dr. Denham by CareFusion [27]. Dr. Denham has denied any wrongdoing and there has been no determination of liability. After the settlement the case is considered closed.

Industrial conflicts-of-interest can also be found in many studies and are almost impossible to eliminate [28]. However, all significant conflicts-of-interest need to be declared. Such was done by one of the authors of the REDUCED MRSA Study, whose institution conducted the study, declaring he was on the speaker’s bureau of “Sage” [8]. The chlorhexidine used in this study was made by Sage Products [8]. Declaring a conflict-of-interest does not imply wrongdoing, but if a significant conflict-of-interest exists, a research study should be carefully scrutinized before being used as a keystone of healthcare policy formulation.

### Changing of research metrics

Concerns of the REDUCED MRSA study also arose because of changes in metrics recorded on www.clinicaltrials.gov more than six months after the trial completion date [29]. Metrics for urinary cultures and central line associated blood stream infections were eliminated and the metric for “Any Pathogens” was added. The latter outcome was reported as statistically significant. The authors responded in a letter [30] to a commentary in Antimicrobial Agents and Chemotherapy (AAC) [3] by stating all secondary outcomes were declared before trial completion and data analysis. However, deletions of metrics after trial initiation should not be allowed, since it may introduce publication bias and any additions in metrics should be clearly explained in the methods section [31]. After publication of the AAC commentary and two years after the study completion date, the metrics were again changed in www.clinicaltrials.gov [32] adding back a metric on urinary tract infections and explaining the data on CLABSI could not be reported due to problems in standardizing the denominator.

To prevent publication bias all metrics that have been defined at trial initiation should be reported and new metrics added should be clearly identified as such in a trial’s methodology section. Since one may pick and choose from multiple possible metrics to add post hoc, the impact of the added metric achieving statistical significance is lessened.

### The use of enhanced hand hygiene as opposed to ADI

Fätkenheuer, et al. [1] correctly states that hand hygiene is a crucial intervention in MRSA control. However, MRSA can also spread by indirect contact and has been observed to live in the environment on commonly used plastics for over 51 days [33]. Within 33 hours, 35% of MRSA colonized patients contaminate their environment [34] and carriers have been reported to be more likely to contaminate the environment than infected patients [35]. Identification of carriers is, thus, imperative.

Although hand hygiene is an indispensable component in MRSA control, it is unlikely that effective control can only be achieved with this intervention alone. This position is supported by Lee, et al. [13], which found implementation of enhanced hand hygiene alone was “not effective” in reducing MRSA.

### Lack of strong standards for control of MDROs in the United States

The recent Ebola epidemic has caused a reevaluation of the standards in the United States for control of all MDROs. We have previously reviewed the history of policy formulation in the United States and concluded that the lack of firm U.S. standards regarding surveillance and isolation may be driven by a desire to avoid the enactment of legislative mandates [3]. Even U.S. standards regarding contact precautions may need to be strengthened. Stating that “a single patient room is ***preferred*** for patients who require Contact Precautions” [36] gives facilities an option not to isolate patients.

The type of personnel protective equipment (PPE) is also not clearly defined or standardized. This is the focus of a major campaign by the National Nurses United who advocate for stronger PPE standards that are not “multiple choice”.

It is obvious that not all contact precautions are the same. There is variability on both the use of isolation and the type of protective gear. PPE that covers every inch of the body and uses N95 respirators will give greater protection than a surgical mask and gloves. Level IV Hazmat PPE is also more expensive, more cumbersome to use and may require specialized training for donning and doffing. The lack of available effective PPE and lax standards regarding which PPE to use may have been an etiological factor in two U.S. healthcare workers developing a hospital acquired Ebola Infection. As pointed out by Edmond, et all, further research and development is needed for optimizing PPE protection of our healthcare workers [37].

## Conclusion

Based on the analysis of the evidence, we believe policy formulation regarding MRSA surveillance can be characterized by what the Union of Concerned Scientist refers to as “Downplaying evidence and playing up false uncertainty” [38]. It can be argued that those studies that cast the greatest doubt on surveillance obtained their negative findings by not having access to timely test results or by not taking effective measures once the results were known. In prominent chlorhexidine bathing studies, a different analysis of the results appears to mitigate the positive results. Some might consider these problems to be below the standard-of-care and the use of these studies in policy formulation to be inappropriate.

The use of surveillance and isolation to address epidemics and common dangerous pathogens should solely depend upon ADI’s ability to prevent the colonization and infection of other patients through indirect contact. One must ask: Why should we treat the MRSA epidemic any differently from the Ebola epidemic? Being a virus, Ebola would be expected to have a much shorter lifespan in the environment than MRSA and, thus, one would expect not spread as easily through indirect contact. We have been asked, how can one possibly compare the two? At the time of this writing we are in the midst of a very dangerous Ebola epidemic with no end in sight. But we should not forget MRSA has cost hundreds of thousands of lives and untold disability. To patients that have been maimed and families that have lost loved ones, there is little difference between the two.
